# Lung cancer gene expression database analysis incorporating prior knowledge with support vector machine-based classification method

**DOI:** 10.1186/1756-9966-28-103

**Published:** 2009-07-18

**Authors:** Peng Guan, Desheng Huang, Miao He, Baosen Zhou

**Affiliations:** 1 Department of Epidemiology, School of Public Health, China Medical University, Shenyang 110001, PR China; 2 Key Laboratory of Cancer Etiology and Intervention, University of Liaoning Province, Shenyang 110001, PR China; 3 Information Center, the First Affiliated Hospital, China Medical University, Shenyang 110001, PR China

## Abstract

**Background:**

A reliable and precise classification is essential for successful diagnosis and treatment of cancer. Gene expression microarrays have provided the high-throughput platform to discover genomic biomarkers for cancer diagnosis and prognosis. Rational use of the available bioinformation can not only effectively remove or suppress noise in gene chips, but also avoid one-sided results of separate experiment. However, only some studies have been aware of the importance of prior information in cancer classification.

**Methods:**

Together with the application of support vector machine as the discriminant approach, we proposed one modified method that incorporated prior knowledge into cancer classification based on gene expression data to improve accuracy. A public well-known dataset, Malignant pleural mesothelioma and lung adenocarcinoma gene expression database, was used in this study. Prior knowledge is viewed here as a means of directing the classifier using known lung adenocarcinoma related genes. The procedures were performed by software R 2.80.

**Results:**

The modified method performed better after incorporating prior knowledge. Accuracy of the modified method improved from 98.86% to 100% in training set and from 98.51% to 99.06% in test set. The standard deviations of the modified method decreased from 0.26% to 0 in training set and from 3.04% to 2.10% in test set.

**Conclusion:**

The method that incorporates prior knowledge into discriminant analysis could effectively improve the capacity and reduce the impact of noise. This idea may have good future not only in practice but also in methodology.

## Background

A reliable and precise classification is essential for successful diagnosis and treatment of cancer. Thus, improvements in cancer classification have attracted more attention [1,2]. Current cancer classification is mainly based on clinicopathological features, gene expression microarrays have provided the high-throughput platform to discover genomic biomarkers for cancer diagnosis and prognosis [3-5]. Microarray experiments also led to a more complete understanding of the molecular variations among tumors and hence to a more accurate and informative classification [6-9]. However, this kind of knowledge is often difficult to grasp, and turning raw microarray data into biological understanding is by no means a simple task. Even a simple, small-scale, microarray experiment generates thousands to millions of data points.

Current methods to help classifying human malignancies based on microarray data mostly rely on a variety of feature selection methods and classifiers for selecting informative genes [10-12]. The ordinary process of gene expression data is as follows: first, a subset of genes with known classification is randomly selected (training set), then, the classifier is trained in the above training set until it is mature, finally, the classifier is used to perform the classification of unknown gene expression data. Commonly employed methods of feature gene selection included Nearest Shrunken Centroids (also known as prediction analysis for microarrays, PAM), shrunken centroids regularized discriminant analysis (SCRDA) and multiple testing procedure(MTP). The conventional methods of classification included k nearest-neighbor classifiers(KNN), linear discriminant analysis(LDA), support vector machine(SVM), back-propagation artificial neural network(BP-ANN) and etc, while the choice of which is a matter of dispute among methodologists [13-15]. So, improvement of existing methods or development of new methods is needed for the analysis of gene expression microarray data. Many gene expression signatures have been identified in recent years for accurate classification of tumor subtypes [16-19]. It has been indicated that rational use of the available bioinformation can not only effectively remove or suppress noise in gene chips, but also avoid one-sided results of separate experiment. However, a relatively few attempts have been aware of the importance of prior information in cancer classification [20-22].

Lung cancer is one of the leading causes of cancer death worldwide [23-26], can be classified broadly into small cell lung cancer (SCLC) and non-small cell lung cancer (NSCLC), and adenocarcinoma is the most common form of lung cancer. Because in China the cigarette smoking rate continues to be at a high level [27], a peak in lung cancer incidence is still expected [28]. Therefore, only lung cancer gene expression microarray dataset was selected in the present study.

In summary, together with the application of support vector machine as the discriminant approach and PAM as the feature gene selection method, we propose one method that incorporates prior knowledge into cancer classification based on gene expression data. Our goal is to improve classification accuracy based on the publicly available lung cancer microarray dataset [29].

## Methods

### Microarray dataset

In the present study, we analyzed the well-known and publicly available microarray dataset, malignant pleural mesothelioma and lung adenocarcinoma gene expression database [29]. This Affymetrix Human GeneAtlas U95Av2 microarray dataset contains 12 533 genes' expression profiles of 31 malignant pleural mesothelioma (MPM) and 150 lung adenocarcinomas (ADCA, published in a previous study [30]), aims to test expression ratio-based analysis to differentiating between MPM and lung cancer. In this dataset, a training set consisted of 16 ADCA and 16 MPM samples.

### Microarray data preprocessing

The absolute values of the raw data were used, then they were normalized by natural logarithm transformation. This preprocessing procedure was performed by using R statistical software version 2.80 (R foundation for Statistical Computer, Vienna, Austria).

### Gene selection via PAM

Prediction analysis for microarrays (PAM, also known as Nearest Shrunken Centroids) is a clustering technique used for classification, it uses gene expression data to calculate the shrunken centroid for each class and then predicts which class an unknown sample would fall into based on the nearest shrunken centroid. Through this process, it can also identify the specific genes that most determine the centroid. The details of PAM method can be found in several published studies [31,32]. Here we adopted ten independent repeats of 10-fold cross-validation (CV) to avoid overlapping test sets. First, the preprocessed dataset was split into 10 subsets of approximately equal size by random sampling, secondly, each subset in turn was used for testing and the remaining 9 subsets for training. The above procedure was repeated 10 times. The error estimates were averaged to yield an overall error estimate. Note that the training set included 100 samples (16290 cases) and the test set included 100 samples (1810 cases) after the above ten independent repeats of 10-fold cross-validation.

### Gene selection via prior biological knowledge

Published studies were collected in the database National Library of Medicine on the web (, Pubmed) from Jan 1^st^, 2000 until March 31^st^, 2009 according to the retrieval strategy of "human lung adenocaicinoma" and published in the journal entitled "Cancer Research". Prior knowledge was viewed here as a means of directing the classifier using known lung adenocarcinoma genes. For the purposes of this study, prior knowledge was any information about lung adenocarcinoma related genes that have been confirmed in literature. Hence, due to the journal's scope and the author's institution's accessibility, we restricted our attention to the journal entitled "Cancer Research". Cancer Research's publication scope covers all subfields of cancer research. The full texts of the papers were downloaded and then lung adenocarcinoma-related genes were retrieved from the literature. Then, after these genes' locations in the original dataset were collected, the genes were tested through multiple testing procedure in the training set provided by Gordon et al [29]. Significant genes were retained after the significant level was set as 0.05 to exclude the non-significant genes.

The combination of the feature genes selected by PAM method and from prior knowledge will be used to direct following classification.

### Classification via modified SVM

**Figure 1:**
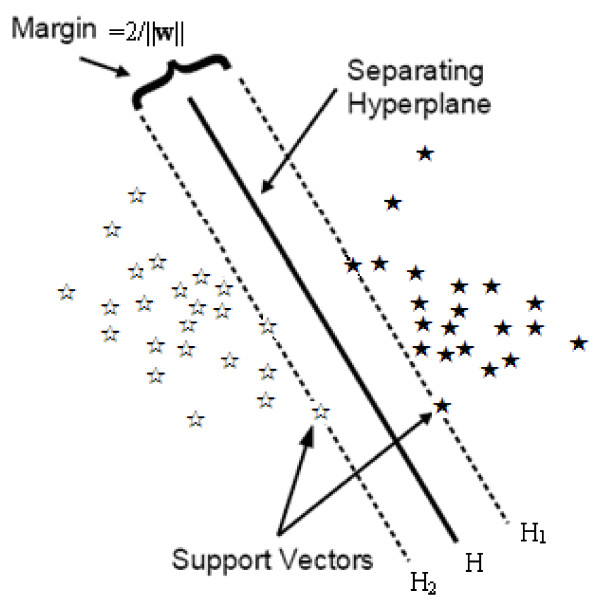
**Classification via SVM (linear separable case)**.

Support Vector Machines (SVM) developed by Cortes & Vapnik [33] in 1995 for binary classification is currently a hot topic in the machine learning theory and one of the most powerful techniques for classification of microarray data. SVM's basic idea for classification may be roughly shown as follows, basically, we are looking for the optimal separating hyperplane between the two classes by maximizing the margin between the classes' closest points (see Figure 1) – the points lying on the boundaries are called support vectors H_1 _and H_2_, and the middle of the margin H is the optimal separating hyperplane. Except for linear decision making, SVM can also solve non-linear problems by first mapping the data to some higher dimensional feature space and constructing a separating hyperplane in this space. Several kernel functions have been introduced in order to deal with non-linear decision surfaces, (1) linear kernel: K(x, y) = x•y; (2) polynomial kernel: K(x, y) = [(x•y)+c]^d^, d = 1, 2, ...; (3) radial basis kernel: K(x, y) = exp{-|x-y|^2^/σ^2 ^}; (4) Sigmoid kernel: K(x, y) = tanh [b(x•y)+c], where b, c and σ are parameters. Among these four types of kernel function, radial basis kernel showed best performance according to the results from similar studies [34,35]. The correct choice of kernel parameters is crucial for obtaining good results, so an extensive search must be conducted on the parameter space before results can be trusted. Here we adopted radial basis kernel function and 5-fold cross-validation in the training set to search the best parameters for SVM-based classification in the test set.

### Evaluation of model performance

**Figure 2:**
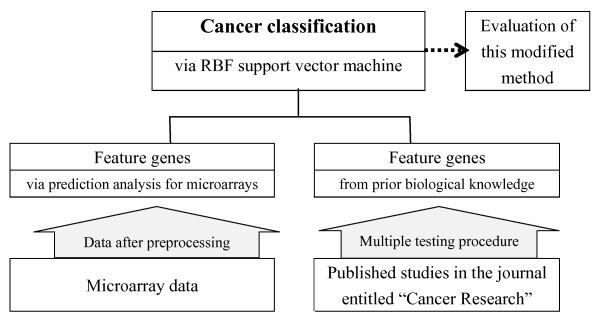
**Framework of our proposed method**.

Classification accuracy and the standard deviations of our proposed method (with prior knowledge) were compared with the original one (no prior knowledge) in the training set and test set. The framework of the above mentioned procedures is shown in Figure 2.

### Statistical analysis

All the statistical analyses were conducted using R statistical software version 2.80 (R foundation for Statistical Computer, Vienna, Austria).

## Results

### Genes selected by PAM

The number of genes selected by PAM method varied from 4 to 12 with an average 7.81, and the standard deviation 2.21. The combination of genes selected by PAM is shown in Table 1. Among them, CEACAM6, calretinin, VAC-β and TACSTD1 appeared in the results all the time.

**Table 1:** Gene lists selected by Prediction Analysis for Microarrays

Gene name	GenBank access No.	Location at HG_U95Av2
ERBB3	M34309	1585_at
CD24	L33930	266_s_at
TACSTD2	J04152	291_s_at
UPK1B	AB015234	32382_at
HIST1H2BD	M60751	38576_at
TITF-1	U43203	33754_at
CLDN3	AB000714	33904_at
CEACAM6	M18728	36105_at
PTGIS	D83402	36533_at
SFTPB	J02761	37004_at
caltrtinin	X56667	37157_at
VAC-β	X16662	37954_at
claudin-7	AJ011497	38482_at
AGR2	AF038451	38827_at
TACSTD1	M93036	575_s_at

### Gene selection via prior biological knowledge

After reviewed the full text of literature, twenty-three lung adenocarcinoma-related genes were selected. Then, Table 2 lists the eight significant genes that passed the multiple testing procedure in the training set provided by Gordon et al. The details of these genes are shown in Table 2.

**Table 2:** Genes as prior biological knowledge

Gene name	GenBank access No.	Location at HG_U95Av2
CXCL1	J03561	408_at
IL-18	U90434	1165_at
AKAP12	X97335	37680_at
KLF6	U51869	37026_at
AXL	M76125	38433_at
MMP-12	L23808	1482_g_at
PKP3	Z98265	41359_at
CYP2A13	U22028	1553_r_at

### Evaluation of model performance

**Figure 3:**
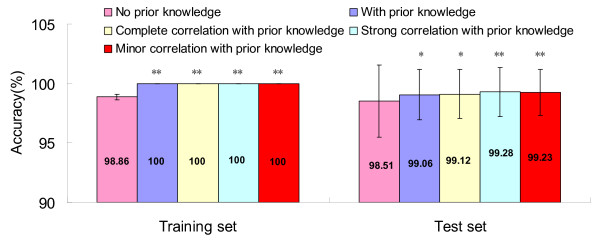
**Accuracy comparisons, no prior knowledge vs. with prior knowledge**. Note: * Accuracy is significantly higher when compared to no prior knowledge at the 0.05 level (2-tailed). ** Accuracy is significantly higher when compared to no prior knowledge at the 0.01 level (2-tailed).

Our proposed method performed better after incorporating prior knowledge (Figure 3). Accuracy of the modified method improved from 98.86% to 100% in training set and from 98.51% to 99.06% in test set. The standard deviation of the modified method decreased from 0.26% to 0 in training set and from 3.04% to 2.10% in test set.

Here, we considered another situation, if there was an overlap between the two sources of genes, i.e. there existed the multi-collinearity, was there any influence on the performance of classification? Hence, taking into account the effect of overlap seemed natural for the current study. Expression quantity of VAC-β with a coefficient 1, 0.5 and 0.05 which meant complete, strong and minor correlation was added to data set for comparison, respectively. The accuracy in the above situation is 99.12%, 99.28%, 99.23% with the standard deviation 2.04%, 2.04%, 1.93%, respectively (Figure 3). McNemar's test was adopted to compare the accuracy between 'no prior knowledge' and the other 4 situations (with prior knowledge, complete correlation with prior knowledge, strong correlation with prior knowledge and minor correlation with prior knowledge) in training set and test set, and all the differences were statistically significant.

The accuracy in the training set was better than that in the test set, and the standard deviations were lower in training set than those in test set. Although Chi-square test indicated that the differences between them were statistically significant, the two sets were not comparable, and the difference may be caused by the large sample size. Training set was used for training and fitting, while test set focused on testing the ability to extrapolate.

## Discussion

Microarrays are capable of determining the expression levels of thousands of genes simultaneously and have greatly facilitated the discovery of new biological knowledge [36]. One feature of microarray data is that the number of tumor samples collected tends to be much smaller than the number of genes. The number for the former tends to be on the order of tens or hundreds, while microarray data typically contain thousands of genes on each chip. In statistical terms, it is called 'large p, small n' problem, i.e. the number of predictor variables is much larger than the number of samples. Thus, microarrays present new challenge for statistical methods and improvement of existing statistical methods is needed. Our research group's interest is lung cancer, we found that one of the key issues in lung cancer diagnosis was the discrimination of a primary lung adenocarcinoma from a distant metastasis to the lung, and so, it was important to identify which contribute most to the classification.

The present study used the combination of the genes selected by PAM and the genes from published studies, the result of this proposed idea was superior to that only rely on the genes selected by PAM. Considered from the methodological point, if the priori knowledge is not contrary to the truth, the incorporation of priori information is able to improve the classification accuracy, at least can not reduce the performance. From the point of accuracy improvement, our result is of concordance with the results of other previous studies [37,38]. It is interesting to compare the list of 15 genes selected by PAM and 8 genes as prior biological knowledge. In the current study, there was no overlap between these two gene lists, but the situation of overlap may be encountered in practice. Several genes may share the same or similar functions, so the existing of correlations among these genes from these two sources should be considered. Our result indicated that after the correlated gene had been added, no decrease of accuracy was found, which meant that there was no need to pay excess attention to the situation that overlapping existed between the information from microarray data and prior information.

One of the main limitations for the present study was how to incorporate prior biological knowledge and where to get it from. The prior biological knowledge in our study was retrieved from the literature, while, with the development of science and technology, huge knowledge will be discovered and reported. The magnitude of prior knowledge may have a certain impact on the results more or less. What information can be used as the truth and which kind of information should be excluded need to be further explored, maybe some experience could be borrowed from evidence-based medicine. On the other hand, the minimum number of predictor genes is not known, which may serve as a potential limitation of the study, and the discrimination function can vary (for the same genes) based on the location and protocol used for sample preparation [39]. The complexity of discriminant analysis and the multiple choices among the available discriminant methods are quite difficult tasks, which may influence the adoption by the clinicians in the future. Although highly accurate, microarray data's widespread clinical relevance and applicability are still unresolved.

## Conclusion

In summary, a simple and general framework to incorporate prior knowledge into discriminant analysis was proposed. Our method seems to be useful for the improvement of classification accuracy. This idea may have good future not only in practice but also in methodology.

## Abbreviations

PAM: prediction analysis for microarrays; SCRDA: shrunken centroids regularized discriminant analysis; MTP: multiple testing procedure; KNN: k nearest-neighbor classifiers; LDA: linear discriminant analysis; SVM: support vector machine; BP-ANN: back-propagation artificial neural network; SCLC: small cell lung cancer; NSCLC: non-small cell lung cancer; MPM: malignant pleural mesothelioma; ADCA: adenocarcinoma; CV: cross-validation.

## Competing interests

The authors declare that they have no competing interests.

## Authors' contributions

PG conceived the study and drafted the manuscript. PG, DH, MH and BZ retrieved and reviewed the literature. PG and BZ attracted funding. All authors contributed to the writing of the final version of this paper.
